# Focal segmental glomerulosclerosis – epidemiology aspects in children and adults

**DOI:** 10.1007/s00467-006-0370-5

**Published:** 2007-02-01

**Authors:** Ronald Hogg, John Middleton, V. Matti Vehaskari

**Affiliations:** 1 Department of Pediatrics, St. Joseph’s Hospital & Medical Center, 222 W. Thomas Rd., Suite 410, Phoenix, AZ 85013 USA; 2 Department of Medicine, Division of Nephrology, Duke University Medical Center, Durham, NC USA; 3 Department of Pediatrics, Division of Nephrology, Louisiana State University, New Orleans, LA USA

**Keywords:** Epidemiology, Focal segmental glomerulosclerosis, Nephrotic syndrome

The histologic features of idiopathic forms of focal segmental glomerulosclerosis (FSGS) were first described by Theodor Fahr in the* Handbuch der speziellen pathologischen Anatomie und Histologie* in 1925 [1]. Over the subsequent eight decades much has been written about the histologic features and clinical characteristics of patients with FSGS, but it is only in recent years that attention has been directed to the incidence and prevalence of the disorder in various populations. This article will provide an overview of the epidemiology of FSGS by reviewing published surveys of renal biopsies, experiences from clinical registries of children with renal insufficiency, and data from the U.S. Renal Data Systems (USRDS).

## Diagnostic considerations

Primary FSGS can present at any age, and it always causes proteinuria. It is most commonly diagnosed in patients with overt nephrotic-range proteinuria, but any proteinuria that is fixed and persistent over several months (i.e., not orthostatic or transient proteinuria) may signal underlying FSGS [2, 3]. Since the diagnosis of FSGS depends on obtaining renal histologic material, the observed incidence and demographics of FSGS in both children and adults depend on the population examined and on the indications used for renal biopsy. The typical findings on renal biopsy in idiopathic FSGS are described elsewhere in this series of articles.

## Incidence and prevalence of FSGS in children

An estimation of the incidence and prevalence of FSGS in children is hampered by the fact that most children with nephrotic syndrome (NS), unlike the majority of adults, are not routinely subjected to renal biopsy. Therefore, prevalence and incidence figures are extrapolated from clinical reports, registries, and renal biopsy materials. Clinical reports often make the presumptive diagnosis of minimal change nephrotic syndrome (MCNS) based on steroid responsiveness [4, 5]. This usually leads to an under-diagnosis of FSGS because up to 15–20% of FSGS patients initially respond to steroids [6]. The largest pediatric registry is that of the North American Pediatric Renal Transplant Cooperative Study (NAPRTCS), which collects data not only on transplant patients but also on dialysis and chronic kidney disease (CKD) patients [6]. Based on results from nearly 6000 children with CKD, in whom the estimated glomerular filtration rate (GFR) was less than 75 ml/min per 1.73 m^2^, it has been shown that FSGS carries the highest likelihood of progressing to end-stage renal disease (ESRD), exceeding the risk of hypoplasia, obstructive uropathy, and reflux nephropathy. According to NAPRTCS data, nearly 60% of such children with a diagnosis of FSGS progress to dialysis or transplantation within 24 months of entry into the registry. However, because reporting is entirely voluntary and involves mainly pediatric centers, there is significant underreporting, and selection bias may exist.

Clinical surveys from North America and the United Kingdom have reported the incidence of NS to be between two and four new cases per 100,000 children per year, with biopsy-confirmed FSGS comprising 15–20% of the total [5, 7–10]. However, as noted above, many children with NS do not undergo a diagnostic renal biopsy unless they are shown to be steroid-resistant. A study from Canada reported an incidence of FSGS of 0.37 to 0.94 new cases per 100,000 children per year [5], and similar results were obtained in the U.S. Midwest [9]. In the NAPRTCS database, patients with FSGS account for 14.2% of dialysis patients and for 11.5% of transplant patients; the differences may reflect a reluctance by some centers to transplant FSGS patients for fear of disease recurrence. Members of the Southwest Pediatric Nephrology Study Group (SPNSG) reported that FSGS was diagnosed in 75 of 1053 (7.1%) patients with NS who underwent a renal biopsy between 1972 and 1981 [11].

While there is a male preponderance among children with MCNS, most reports have described no clear gender difference in patients with FSGS [4, 11, 12], although in the SPNSG series, all 18 children with FSGS below the age of 3 years were boys [11].

Ethnicity and age appear to play important roles in the prevalence of FSGS. The NAPRTCS and SPNSG reports include approximately equal numbers of Caucasian and African American children with FSGS despite the large difference in the racial distribution of the underlying populations [6, 11]. According to the NAPRTCS report, FSGS is the most common cause of ESRD in African-American children in that cohort, accounting for 23% of all pediatric patients with ESRD. In Caucasian children, FSGS is only the third most common primary disease and is responsible for approximately 10% of all pediatric patients with ESRD [5]. In contrast, African-American children with NS appear to be more likely to have FSGS than MCNS [4, 10, 13]. Whether the frequency of FSGS in Hispanic children differs from that in Caucasians is unclear [14, 15].

The prevalence of FSGS among all children with NS who undergo a renal biopsy increases with age. However, it is not clear if the true population-based incidence of FSGS is age-dependent. Two thirds of children with NS present before the age of 6 years, but only a minority of the younger children exhibit FSGS, whereas FSGS is the most common histology in the older group. The frequency of FSGS in NS patients presenting before 6 years of age is less than 10%, but increases to 20–50% or more in patients presenting in adolescence [4, 9–11, 16]. The combined effect of age and ethnicity is responsible for the reported high frequency of FSGS in kidney biopsies from adolescent African-American patients with NS [4, 10, 13]; both age and ethnicity are probably independent risk factors for FSGS histology.

An intriguing recent development has been the apparent increase in the incidence of FSGS in children [4, 5, 10, 17]. Differences in the ages and ethnicity of the subjects do not fully explain these findings. Filler et al., for instance, studied mostly Caucasian children in Canada and found that the calculated incidence of FSGS increased from 0.37 to 0.94 per 100,000 children per year from the first to the second half of a 17-year period between 1985 and 2002 [5]. The increase appears to be present mostly in older children [4]. Similar increases in incidence have not been seen in other types of NS in children [4, 5]. The cause of the increased incidence is not known but is unlikely to be due to genetic factors. The role of environmental pollution has been proposed but remains hypothetical [18]. Morbid obesity-associated FSGS has increased in renal biopsy material [19], but because it remains uncommon, the current adult and pediatric obesity epidemic probably does not explain the increase in FSGS.

## Incidence and prevalence of FSGS in adults

Several publications suggest that the prevalence of idiopathic FSGS in adults may also be increasing [20, 21]. Observations from two metropolitan centers suggest that FSGS was found in 2.5–4% of native renal biopsies in the 1970s, but in 12.2–18.7% in this decade, making FSGS the most common diagnosis based on native kidney biopsies. FSGS becomes less common with advancing age, but it is still a significant problem in the elderly. In a series of 1368 renal biopsies from patients over 60 years of age, FSGS was present in 5.4% of those patients with nephrotic syndrome [20]. These trends in increased numbers of FSGS cases are observed in both African Americans and Caucasians and occurred despite stable or falling rates of other glomerular diseases, such as minimal change disease and membranous nephropathy [21].

Two large series in adults support the fact that primary FSGS is a risk factor for developing ESRD. In one report of outcomes from immunosuppressive therapy in 59 adult patients with FSGS, 30% of patients developed some level of renal insufficiency within 5 years [22, 23]. Wehrmann and colleagues reported results from 250 patients with idiopathic FSGS (average age: 32 years) in which they found an overall 10-year renal survival of 67% [23]. The risk of developing ESRD in idiopathic FSGS can be predicted at the time of diagnosis. Heavy proteinuria, either at the time of biopsy but particularly following treatment, portends a poor long-term outcome. Other features that increase the risk for progressive renal disease include elevated serum creatinine at the time of diagnosis, hypertension, age at time of diagnosis, and male gender [22, 24, 25]. The most compelling predictor for progression of FSGS, however, appears to be the response of proteinuria after treatment is initiated. One series suggests that if a patient with primary FSGS fails to respond to initial therapy (most often steroid-based), the likelihood of renal death is as great as if the patient received no treatment at all [25]. However, a full assessment of relative risk will require larger cohorts of patients with FSGS who received consistent interventions.

The risk of FSGS-related ESRD in adults is also supported by data published by the USRDS [26]. Documented FSGS accounts for more than 7000 patients currently receiving ESRD therapy in the United States, and it is likely that primary FSGS accounts for at least some of the 25,000 patients with unspecified forms of glomerulonephritis, the 100,000 patients with ESRD attributed to hypertension, and the 20,000 patients with an unknown cause of ESRD [26]. The USRDS also demonstrates a dramatic increase in the incidence of ESRD due to FSGS, particularly over the last decade in the African-American population. In the Caucasian U.S. population, new-onset ESRD due to FSGS has slowly increased to a rate of approximately five cases/million population in 2003. In contrast, the incidence has increased nearly five-fold in the African-American population since the early 1980s to 30–40 cases per million population [26].

## Familial FSGS

Occasional cases of familial FSGS have long been recognized. In 1973 Habib suggested that 12% of all pediatric cases of FSGS had an affected sibling [2]. Although most cases of FSGS are sporadic with unknown pathophysiology, it is now clear that mutations in structural podocyte-associated proteins account for a portion of familial cases and for at least some of the sporadic cases. A mutation in the *NPHS2* gene, which encodes the protein podocin, has been linked to autosomal-recessive steroid-resistant nephrotic syndrome in children [27]. However, there are large differences between different ethnic populations. For example, approximately one third of Israeli-Arab children but none of Israeli-Jewish or Japanese children with sporadic FSGS were reported to have *NPHS2* mutations [27, 28]. The frequency of *NPHS2* mutations in North American children is not known. Mutations in the a-actinin 4 gene (*ACTN4*) have been associated with autosomal-dominant FSGS in adults, a disease characterized by a variable risk of progression to ESRD. Most recently, autosomal-dominant FSGS has been linked to the gene that encodes the transient receptor potential cation channel, subfamily C, member 6 (*TRPC6*) [29]. Although more data are needed, it is likely that mutations in the known FSGS causative genes account for only a minority of cases of FSGS in children and adults. A more complete review of the genetic aspects of FSGS is provided elsewhere in this series of articles.

## Conclusion

It is clear that there is still much to learn about the epidemiology of idiopathic FSGS. However, there are encouraging signs that the body of knowledge will be enhanced in coming years by the various registries and population-based studies currently underway.

## Questions

(Answers appear following the reference list)

1. Which of the following statements is correct?
  - Patients with idiopathic FSGS often present with microscopic hematuria without proteinuria.
  - All patients with FSGS have nephrotic range proteinuria.
  - Patients with FSGS have proteinuria of varying severity.
  - None of the above.
2. Which of the following carries the highest risk for a patient with FSGS to progress to ESRD?
  - Male gender.
  - Persistent proteinuria after therapy.
  - Hyperlipidemia.
  - Caucasian race.
  - Age.
3. Which of the following is incorrect?
  - Accurate data concerning the outcome of patients with FSGS is lacking since the disease was first described only 30 years ago.
  - The incidence of the disease appears to be decreasing in the last 20 years.
  - Most cases of FSGS can now be linked to specific gene mutations that disturb podocyte function.
  - All of the above.
  - None of the above.
4. Which of the following is correct?
  - FSGS is a more frequent cause of ESRD in Caucasian children than in African-American children.
  - FSGS is more commonly associated with nephrotic syndrome in Hispanic children than in white children.
  - Children with FSGS account for less than 10% of African-American children who progress to ESRD.
  - All of the above.
  - None of the above.
